# cWords - systematic microRNA regulatory motif discovery from mRNA expression data

**DOI:** 10.1186/1758-907X-4-2

**Published:** 2013-05-20

**Authors:** Simon H Rasmussen, Anders Jacobsen, Anders Krogh

**Affiliations:** 1 Bioinformatics Centre, Department of Biology, University of Copenhagen, Ole Maaløes Vej 5, Copenhagen N, 2200, Denmark; 2 Computational Biology Center, Memorial Sloan-Kettering Cancer Center, New York, NY, 10065, USA

**Keywords:** MicroRNA, siRNA, RNA binding proteins, Motif discovery, Post-transcriptional regulation

## Abstract

**Background:**

Post-transcriptional regulation of gene expression by small RNAs and RNA binding proteins is of fundamental importance in development of complex organisms, and dysregulation of regulatory RNAs can influence onset, progression and potentially be target for treatment of many diseases. Post-transcriptional regulation by small RNAs is mediated through partial complementary binding to messenger RNAs leaving nucleotide signatures or motifs throughout the entire transcriptome. Computational methods for discovery and analysis of sequence motifs in high-throughput mRNA expression profiling experiments are becoming increasingly important tools for the identification of post-transcriptional regulatory motifs and the inference of the regulators and their targets.

**Results:**

cWords is a method designed for regulatory motif discovery in differential case–control mRNA expression datasets. We have improved the algorithms and statistical methods of cWords, resulting in at least a factor 100 speed gain over the previous implementation. On a benchmark dataset of 19 microRNA (miRNA) perturbation experiments cWords showed equal or better performance than two comparable methods, miReduce and Sylamer. We have developed rigorous motif clustering and visualization that accompany the cWords analysis for more intuitive and effective data interpretation. To demonstrate the versatility of cWords we show that it can also be used for identification of potential siRNA off-target binding. Moreover, cWords analysis of an experiment profiling mRNAs bound by Argonaute ribonucleoprotein particles discovered endogenous miRNA binding motifs.

**Conclusions:**

cWords is an unbiased, flexible and easy-to-use tool designed for regulatory motif discovery in differential case–control mRNA expression datasets. cWords is based on rigorous statistical methods that demonstrate comparable or better performance than other existing methods. Rich visualization of results promotes intuitive and efficient interpretation of data. cWords is available as a stand-alone Open Source program at Github https://github.com/simras/cWords and as a web-service at: http://servers.binf.ku.dk/cwords/.

## Background

MicroRNAs (miRNAs) are endogenous small regulatory RNAs of size approximately 22 nucleotides. miRNAs, bound by the RNA induced silencing complex (RISC), repress gene and protein expression post-transcriptionally. miRNA targeting and binding of complementary messenger RNA (mRNA) sequences - often in the 3′ untranslated regions (UTRs) - generally leads to target mRNA degradation [1-3]. Perfect base-pairing between nucleotide 2 to 8 of the mature miRNA (the seed) and the mRNA target site plays an essential role [3], but cannot alone explain the full regulatory potential of miRNAs [4].

The function of a miRNA in a given cellular context can be studied experimentally by analyzing changes in mRNA expression after miRNA inhibition [5,6] or overexpression [1,2]. When interpreting data from such experiments it is important to establish that the miRNA was successfully and efficiently perturbed leading to change in expression of target mRNAs. This can be achieved by showing differential regulation of the predicted target mRNAs [2] or by showing seed site enrichment using unbiased 3′UTR motif analysis of differentially expressed genes [7-10]. An unbiased motif analysis may have additional advantages as a standard tool when analyzing miRNA perturbation experiments. For example, miRNA target prediction methods may not detect non-canonical target motifs specific to the perturbed miRNA, and systematic analysis of miRNA perturbation experiments has shown that in addition to miRNA seed sites, other 3′UTR motifs, some corresponding to known binding sites of RNA binding proteins (RNA-BPs), can also be predictive of the observed mRNA expression changes [7]. There is therefore a need for computational methods that allow for unbiased and systematic analysis of mRNA sequence motifs in miRNA perturbation experiments to confirm effective experimental perturbation and to explore regulatory sequence elements other than established miRNA binding sites.

Motif discovery has a long history in bioinformatics [11], in particular for analysis of transcription factor binding sites [12]. There are many different approaches to motif discovery. Most use a fixed set of sequences and identify motifs that are overrepresented in this set compared to a Markov chain background model (Gibbs Sampler [13], MEME [14], and Weeder [15]). Other methods do discriminative analysis, where the goal is to identify motifs that are over-represented in a positive set compared to a negative or background set of sequences (DEME [16] and [17]). However often we are dealing with transcriptome-wide measurements of gene expression, and *a priori* it is difficult to set a natural cut-off that defines the positive (or negative) set.

Recently, methods for identifying correlations of word occurrences in mRNA sequences and transcriptome-wide changes in gene expression have been developed. miReduce [8] and Sylamer [9] are two such methods designed for unbiased analysis of miRNA regulation in mRNA 3′UTR sequences (and for analyses of other types of gene regulation). miReduce uses a stepwise linear regression model to estimate the words that best explain the observed gene expression changes. Sylamer computes word enrichment based on a hyper-geometric test of word occurrences in a ranked list of sequences. Sylamer is computationally efficient and allows for bin-wise 3′UTR sequence composition bias correction.

Here we present cWords, a method for correlating word enrichment in mRNA sequences and changes in mRNA expression. It permits for correction of sequence composition bias for each individual sequence and is based on methods developed in [7]. By development of robust and efficient parametric statistics, cWords offers a factor 100 to 1000 speed gain over the previous permutation-based framework. An exhaustive 7mer word analysis of a gene-expression dataset can be completed in less than 10 minutes mainly due to efficient approximations of statistical tests, and the parallelized implementation that enables full utilization of multicore computer resources.

cWords includes methods for clustering and visualization of enriched words with similar sequences that can aid exploratory analysis of enriched words and degenerate motifs such as noncanonical miRNA binding sites and RNA-BP binding sites. We show that cWords is effective for analyzing miRNA binding and regulation in miRNA overexpression and inhibition experiments, and we demonstrate how cWords can be used to identify enrichment of other types of regulatory motifs in such experiments. We demonstrate that miReduce, Sylamer, and cWords exhibit comparable performance on a panel of miRNA perturbation experiments. Finally, we demonstrate how cWords can be used to identify potential siRNA off-target binding and regulation in RNAi experiments, and to discover endogenous miRNA binding sites in an experiment profiling mRNAs bound by Argonaute ribonucleoprotein.

## Results and discussion

We have developed an efficient enumerative motif discovery method that can be used for extracting correlations of differential expression and motif occurrences. In brief, sequences are ranked by fold change of expression, and motifs (words) are correlated with gene ranks. Unlike other methods, cWords can detect subtle correlations of words only present in few sequences due to sequence specific background models. The rigorous statistical framework allows for simultaneous analysis of multiple word lengths, and words are clustered into motifs presented in plots providing both overview and in-depth information for interpretation.

### The summary plots of cWords

**Figure 1:**
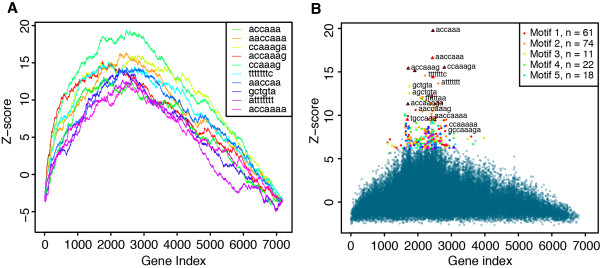
**Enrichment of miR-9 seed sites in 3′ end untranslated regions (3′UTRs).** (**A**) Enrichment profile for the top ten enriched words in 3′UTRs after miR-9 transfection. Each line represents the running sum over all scores that quantify degree of enrichment according to gene downregulation (from most downregulated to most upregulated). miR-9 seed sites (7mer-m8 seed ACCAAAG) and similar words dominate the top ten regulatory words predicted. (**B**) Word cluster plot showing words in 3′UTRs ranked by differential expression, after miR-9 transfection. Each dot represents a word, summarizing Z-scores, and enrichment specificity indices of the enrichment profiles of negatively correlated 6, 7 and 8mer words. Triangles annotate known seed sites of human miRNAs. Triangles with a black border show miR-9 seed sites. Words are clustered by sequence similarity using the UPGMA algorithm and colored according to what motif (or cluster) they belong to (only some among top 100 words are plotted).

cWords provides different summary visualizations to aid in interpretation of a word correlation analysis. The enrichment profile plot is a visualization of the cumulative word enrichment (a running sum graph) across the sorted list of gene sequences. This plot is similar to the plots of Gene Set Enrichment Analysis [18] and Sylamer [9], and it provides a detailed view of enrichment as function of gene expression change for a specific word. Figure 1A shows an example of an enrichment profile plot for the words most significantly enriched in genes downregulated after miR-9 overexpression in HeLa cells [2].

The gene rank in the enrichment profile plot at which the global maximum enrichment score is obtained is termed the enrichment specificity (ES) index. A low ES index is indicative of a specific enrichment signal corresponding to enrichment of a motif in a small set of strongly differentially expressed genes. Oppositely, a high ES index reflects that the word enrichment was found for a larger set of less differentially expressed genes. Words enriched in sets of genes with a large intersection will tend to exhibit similar enrichment profiles and have ES indices that are numerically close. For example, variants of miRNA target sequences (seed sites with 1 or 2 nucleotides offsets) tend to have similar ES indices when analyzing miRNA overexpression experiments (Figure 1A).

The enrichment profile plot provides a lot of detail for individual words, but is also limited by the number of words that can be effectively summarized in the same plot, which may be an important factor in the discovery phase of a motif analysis. For this purpose we developed the word cluster plot (Figure 1B). This plot shows the maximum enrichment score versus the ES index for all words, and it displays word relationships found through word similarity clustering. We found that this type of plot produces a simple and yet informative summary for miRNA perturbation experiments. For example, when analyzing expression changes after miR-9 overexpression in HeLa cells, the word with strongest enrichment in 3′UTRs of downregulated genes corresponds to the 7mer seed site of miR-9 (Figure 1B). Several shifted variants of the seed site also show enrichment in the plot highlighting the preference for sites with a flanking adenosine. Furthermore, the plot reveals significant enrichment for certain T-rich motifs (including TTTTAAA, DNA-alphabet was used with T instead of U), which were also reported in our previous study [7]. The word cluster plot can therefore provide a rich and unbiased summary for exploration of regulatory motifs associated with gene expression changes.

### cWords analysis of miRNA target sites in coding regions of mRNAs

**Figure 2:**
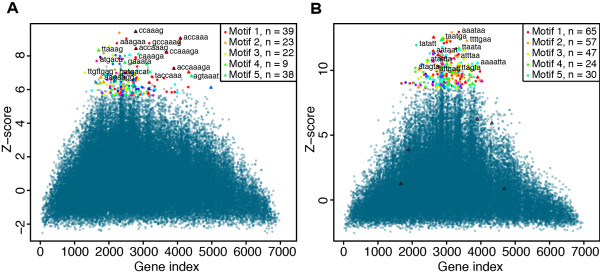
**Enrichment of miR-9 and miR-128 seed sites in coding regions.** (**A**) Word cluster plot shows enriched 6, 7 and 8mer words in coding regions after miR-9 transfection. The word cluster plot is described in Figure 2B. (**B**) Word cluster plot shows enriched 6, 7 and 8mer words in coding regions after miR-128 (7mer-m8 seed CACTGTG) transfection.

Analyses of target site efficacy in miRNA perturbation experiments and target site evolutionary conservation have shown that target sites in mRNA coding sequences (CDS) exist but are much less effective and frequent compared to sites in 3′UTRs [3], and for this reason target sites in CDS are often not included in target prediction databases and likewise frequently ignored in functional analysis. However, miRNA target sites in CDS may be more important for specific miRNAs ([19]) or under certain conditions, and we explored if cWords could be used as a method to evaluate the efficacy of CDS target sites in miRNA perturbation experiments. For analysis of CDS motifs we used a tri-nucleotide background model to correct for differences in codon usage between individual mRNAs. We found highly significant enrichment of miRNA seed sites in CDS of downregulated mRNAs in 8 out of 11 miRNA overexpression experiments (6, 7 or 8mer seed significant and present in top ten words). In Figure 2 we show word cluster plots for cWords analyses of words enriched in CDS of downregulated genes following overexpression of two different miRNAs. In Figure 2A we see that miR-9 is significantly more enriched in 3′UTRs (Figure 1A), but the seed site signal definitely stands out in CDS too. This suggests that miR-9 binding in CDS contributed to the depression of expression levels performed by miR-9, after it was overexpressed. Contrary, miR-128 does not seem to be regulating its targets strongly through binding in CDS (see Figure 2B), the highest ranked seed site is the 7mer A1 site ACTGTGA and it ranked 1,521 (marked by black triangles in the word cluster plot) and the enrichment profile shows no particular over-enrichment in the most downregulated genes. This illustrates that enrichment of seed site signal in CDS varies more than in 3′UTRs. Enrichment analysis is a way to elucidate such differences, which make way for understanding the biological context of the experiment. Data used in the above is described in Supplementary methods in Additional file 1.

### cWords identifies siRNA off-target effects

**Figure 3:**
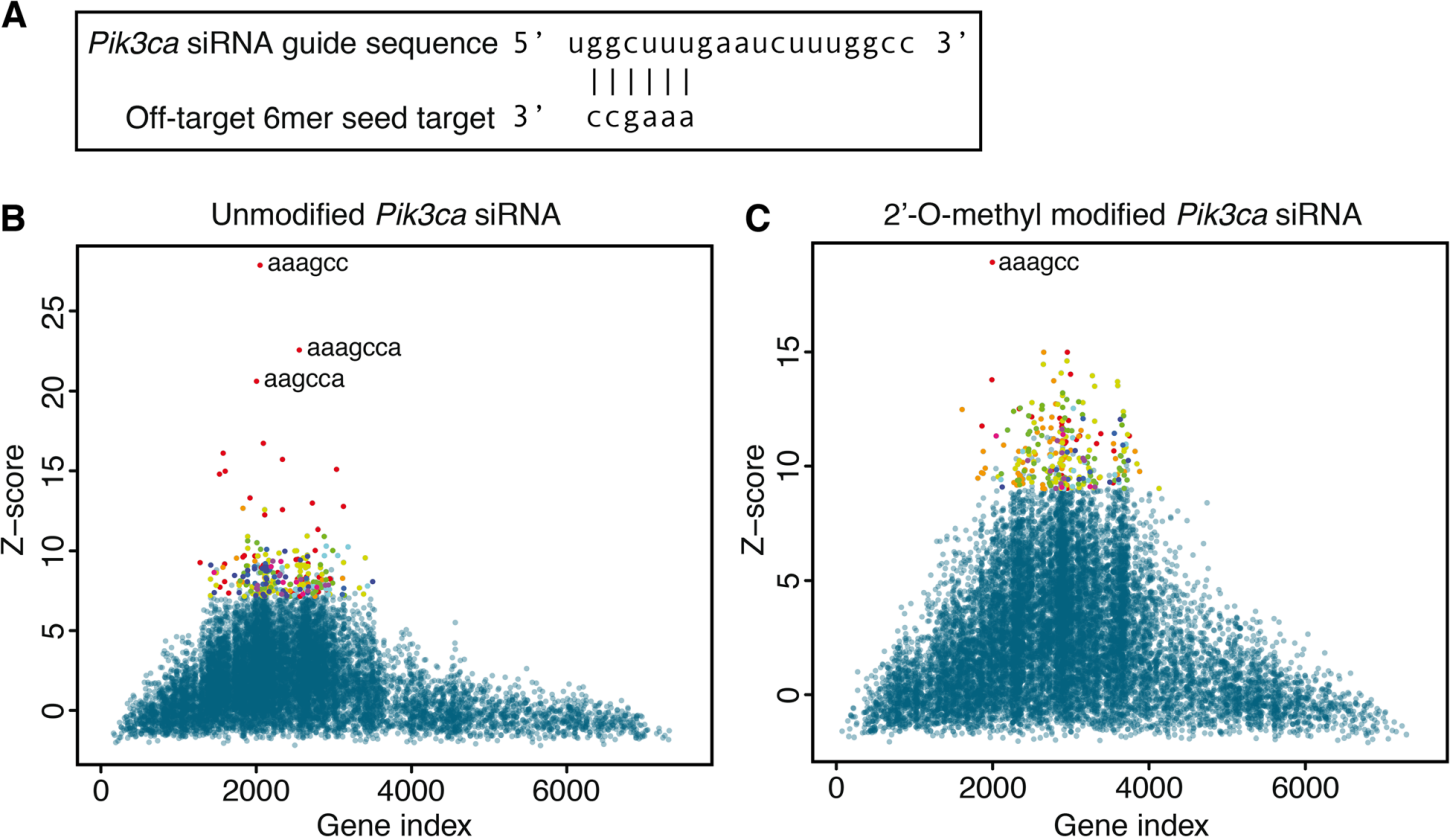
**Modified and unmodified siRNA off-target effects.** cWords word cluster plots showing 6, 7, and 8mer words enriched in 3′UTRs of genes downregulated by siRNA off-target binding after transfection of an unmodified (**B**) and 2-O-methyl modified (**C**) siRNA targeting *Pik3ca*. Enrichment of the 6mer seed (bases 2–7, AAAGCC) of the siRNA is highlighted in bold letters and seed region is illustrated in panel (**A**).

Small interfering RNAs (siRNAs) are double-stranded RNA molecules that can be designed to induce RNAi-mediated cleavage of intended target mRNAs by full complementarity of the siRNA guide strand to the mRNA. However, transfected siRNAs have also been shown to cause unwanted miRNA-like binding, termed off-target effects, where hundreds of mRNAs are destabilized by base pairing to the seed region of the siRNA [7,20-24]. A study has demonstrated that chemical modification of the siRNA can effectively reduce such off-target effects [25]. In this study ten different siRNAs were transfected in HeLa cells in both an unmodified and modified form containing 2-O-methyl modifications in position 2 of the guide strand and positions 1 and 2 of the passenger strand. We used cWords to analyze words enriched in 3′ UTRs of genes downregulated following transfection of the unmodified and modified versions of three siRNAs designed to target *Pik3ca*, *Prkce*, and *Vhl*. For all three siRNAs we found that transfection of the unmodified siRNA resulted in strong enrichment of seed words in downregulated mRNAs (Z-score >14 and seed was ranked 1 of all 6, 7 and 8mers). This effect was notably reduced with the modified *Prkce* and *Vhl* siRNAs (Z-score <6 and seed was not among top 300 words). However, the modified *Pik3ca* siRNA also showed fairly strong seed enrichment in downregulated mRNAs (Z-score = 19.2, rank 1, Figure 3C), but weaker than the unmodified siRNA, Figure 3B. This result suggests that the effect of 2-O-methyl modifications may be dependent on the siRNA sequence and demonstrates how cWords can be a useful tool to identify and diagnose off-target effects in siRNA experiments. The data used in this case is described in Supplementary methods in Additional file 1.

### cWords analysis of endogenous miRNA binding sites in HEK293 cells

**Figure 4:**
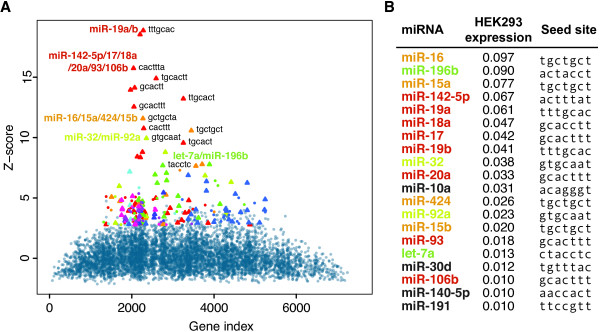
**Word enrichment of Argonaute bound mRNAs.** (**A**) cWords word cluster plot showing 7mer 3′UTR words correlated with Argonaute binding in HEK293 cells. The top ten words are annotated with identifiers of the most abundant (top 20) miRNAs in HEK293 cells when the word and miRNA seed sequence (6 or 7mer) are complementary. (**B**) List of the 20 most abundant miRNAs in HEK293 cells, listing expression (relative clone frequency) and seed site (position 2 to 8) for each miRNA.

Which mRNAs miRNAs target can also be explored without the use of small RNA perturbations. One such experimental technique is relative quantification of mRNAs bound by Argonaute (AGO) proteins. To analyze if cWords could be useful in analysis of such datasets, we used a previously published dataset using HEK293 cells, measuring mRNA abundance in immunoprecipitated (IP) AGO ribonucleoprotein particles (RNPs) relative to background mRNA expression [26]. By sorting mRNAs by relative abundance in AGO IP RNPs, cWords was used to identify 3′UTR words significantly correlated with AGO mRNA binding. Using previously published miRNA expression data from HEK293 cells [27], we found that the top ten words strongest correlated with AGO binding were all complementary to seeds of the most abundant miRNAs in HEK293 cells (Figure 4). This result suggests that cWords can also be a useful tool to study miRNA regulation in assays other than miRNA perturbation experiments. Data used is described in more detail in Supplementary methods in Additional file 1.

### Comparison to miReduce and Sylamer

The performance of cWords was compared to two other methods, miReduce and Sylamer, on the task of identifying seed site binding in mRNA 3′UTRs in a panel of 18 miRNA transfection experiments and one miRNA inhibition.

miReduce uses a stepwise linear regression estimation procedure and does not compute scores for all words of a given length - only the most significant word among a group of strongly correlated words will be included in the model and summarized in the output. Words of different lengths cannot be compared by the Sylamer statistic. Due to these issues we compare performance of the three methods by computing enrichment for all 7mers in each miRNA perturbation experiment. We report the rank of the highest ranking word that is identical to the reverse complement of the canonical A1 7mer seed (identity in positions 2 to 7, with preferentially an A in position 1) or the canonical m8 7mer seed (identity in positions 2 to 8) of the transfected miRNA [3].

For miReduce, Sylamer and cWords, we found that the top-ranked word corresponded to the seed site of the perturbed miRNA in 13 of the 19 experiments [see Table 2 in Additional file 1]. In six experiments the results diverged. For transfection of miR-133a, the top word had an overlap of the six rightmost characters with the six leftmost in the m8 7mer canonical seed site, for all methods. This most likely reflects the biological reality that miRNAs under certain conditions bind in atypical ways. Another exception was in the inhibition of miR-21 [5], where Sylamer ranked a 7mer seed site as number 12 and all higher ranking words were not similar to the seed site. In the other four experiments Sylamer did not rank a 7mer seed site as the first word.

This serves as a demonstration that the three methods are able to find and discriminate the seed motif in datasets where this is expected to be the strongest signal. In five cases cWords performed better than Sylamer, but generally the performance of the three methods was very similar under these benchmarking conditions. The parallel implementation is an advantage of cWords over the other methods. Using four cores cWords finished an analysis on average approximately five times faster than Sylamer and approximately two times faster than miReduce and using 40 cores cWords was up to 20 times faster. In both cases, Sylamer was run disabling approximations to not compromise precision. If a larger window size is used, Sylamer is faster than the other methods. For more details on which data was used in the comparison see Supplementary methods in Additional file 1.

## Conclusions

We have presented cWords, which finds overrepresented words in sets of DNA (or RNA) sequences. Contrary to most other methods, it uses a sensitive statistics that takes the individual sequence composition into account. cWords can rank words across different word lengths and uses clustering to group similar words. cWords outputs multiple summary plots and tables, which in combination provide both an overview and detailed information for in depth analysis of the results.

cWords is designed for analysis of experiments in which gene expression is measured after perturbation of a miRNA. We have shown cWords successfully identifies seed sites as the highest-ranking words in such experiments. Furthermore, we have shown that cWords can identify likely off-target effects of siRNAs mediated by miRNA-like binding of 3′UTRs, and that binding motifs of endogenous miRNAs can be identified from Argonaute immunoprecipitation data.

We conducted a comparative study of cWords, miReduce and Sylamer on published datasets from 19 miRNA transfection and miRNA knockdown experiments. No single method was notably better than the others, and overall the performance of cWords, miReduce, and Sylamer was very good for the specific application of identifying seed sites as high-ranking motifs.

The word cluster plot of cWords provides a summary and a way to associate words among the highest-ranking words. An advantage of both miReduce and cWords is that they can statistically evaluate and compare enrichment for motifs of different lengths. Sylamer can only be used for words of the same length in an analysis and results from analyses of different word lengths are not directly comparable. Sylamer is a fast tool, but actually this is only the case when a large ‘window size’ is used, however, the speedup resulting from a large window size comes at the expense of a less precise background model.

We have strived to make cWords user friendly, and it offers the flexibility of a downloadable Open Source program rich in features as well as the simplicity and ease of use of the cWords web server.

## Methods

cWords is an exact method, in which all words of a given length are counted in the sequences. Based on these word frequencies, enrichment scores (scores of over-representation) are calculated for each word in each sequence by a binomial model with a kth-order Markov Model that corrects for composition bias in each sequence. Enrichment scores are summarized and enrichment profiles normalized in a Kolmogorov like statistics used for ranking and discriminating regulatory words from non-regulatory.

### Scoring word overrepresentation in individual sequences

Whether a word is over-represented is tested in a binomial model with a mono-, di- or tri-nucleotide background estimated for each individual sequence in the following way. The background probability of the word *W* in a given sequence *s* is approximated by a Markov Model of order *k*:

(1) $$P_{k}\left(W\right)=\mu \left(w_{1}\ldots w_{k}\right)\prod_{i=1}^{l_{W}-k}\pi \left(w_{i+k}\left|w_{i},\ldots ,w_{i+k-2},\right)w_{i+k-1}\right)$$

Here *μ*(*w*_1_…*w*_*k*_) is the frequency of the *k* first nucleotides of *W*, *l*_*w*_ is the length of *W* and the product is the probability of the rest of the word given the distribution of (*k* + *1*)-mer words in the sequence [28]. *n* = *l*_*s*_ + *l*_*W*_ + *1* is the number of possible matches in sequence *s*, where *ls* is the length of *s*. Assuming that words occur independently of one another, the probability of a word occurring *m* times or more can be calculated from the binomial distribution function.

(2) $$P(q\geq m|n,p)=\overset{n}{\underset{i=m}{\sum }}\left(\begin{array}{c} n\\ i \end{array}\right)p^{i}{\left(1-p\right)}^{n-i}$$

where *p* = *P*_*k*_(*W*) is the probability of observing *m* occurrences of the word *W* in a sequence (calculated by equation 1). In the original implementation of cWords the expected frequency of a word in a sequence was estimated by shuffling it. The above probability was calculated as the fraction of shuffles where *m* or more instances of the word would occur.

### Evaluating word enrichment in a ranked list of sequences

We consider *u* sequences ranked according to degree of differential expression in increasing order. For each word we calculate a *P* value as described in equation 2. This gives

$$\left\{p_{1},p_{2},\ldots ,p_{u}\right\}$$

which follows the same ordering as the sequences. From these we calculate log scores.

$$ls_{i}=-\ln\left(p_{i}+\alpha \right)$$

where a small number, *α*, is added to regularize very small probabilities (we use *α* = 10^-5^). Using $\overset{-}{\mathrm{ls}}$ to denote the mean of the log-scores over all sequences, we define a running sum of log-scores

$$r_{0}=0$$

$$r_{i}=r_{i-1}+ls_{i}-\overset{-}{\mathrm{ls}},1\leq i\leq u$$

**Figure 5:**
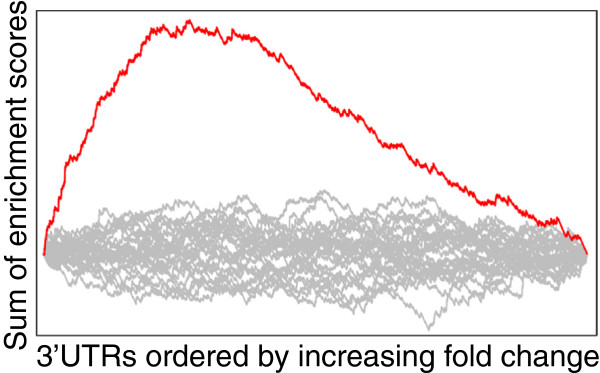
**Word enrichment profile on a background of random permutations.** The red line shows the running sum of log-scores for a specific word and the random permutations of the scores form a background distribution (grey lines). The x-axis represents gene ranks, from most downregulated to most upregulated, the y-axis the cumulative enrichment score.

In Figure 5 a line plot (the red line) of a running sum for a specific word is shown along with running sums for random permutations of the log-scores of the same word (the grey lines). To quantify how much a word deviates from random, we calculate the absolute maximum of the running sum,

$$D=\max_{k}\left(r_{k}\right)$$

In [7], the expected distribution of *D* was computed from permutations of the log-scores. This approach is computationally intensive, and here we provide an efficient analytical solution. Suppose that we do random permutations of the log-score set (as illustrated by the grey lines in Figure 5). Since the running sum starts and ends at *0*, this corresponds to a Brownian bridge, which is a one-dimensional Brownian Motion conditioned on having the same origin and terminal Y-coordinate ([29]). The theoretical distribution of the absolute max of a Brownian bridge is known as the two-sided Brownian Bridge max distribution. It is also known as the Kolmogorov distribution in a slightly different form ([30,31]). A *P* value for some observed value of *D* can be calculated from the distribution function

(3) $$P\left(Y\geq \frac{D}{\sigma }\right)\approx 1-\overset{\infty }{\underset{h=-\infty }{\sum }}{\left(-1\right)}^{h}e^{\frac{-2D^{2}h^{2}}{\sigma^{2}t}},\frac{D}{\sigma }\geq 0,t,\sigma >0$$

where *σ* is the standard deviation of the distribution of log-score *ls*_*i*_ and *t* is the number of genes in the analysis. The above formulation requires that the log-score set has standard deviation 1. To evaluate *D* we need to normalize with the standard deviation *σ* of the complete log-score set for the word in question. This is very similar to the Kolmogorov-Smirnov statistics used for the normalized enrichment scores in Gene Set Enrichment Analysis (supporting text of [18]). We derived moment estimates for *Y*.

$$E\left(Y\right)=\sqrt{\frac{\mathrm{\pi t}}{2}}\left(\ln\left(2\right)\right)$$

$$\mathrm{Var}\left(Y\right)=t\frac{\pi^{2}}{12}-E{\left(Y\right)}^{2}$$

For all words to be comparable we can now calculate the Kolmogorov like statistics

$$Z=\frac{\frac{D}{\sigma }-E\left(X\right)}{\sqrt{\mathrm{Var}\left(X\right)}}$$

*X* = *σY* is the observed enrichment score distribution, *Z* is approximately normally distributed and these *Z*-scores are used for ranking words in cWords.

### Clustering words into motifs

Signals of regulatory sites typically surface as degenerate motifs and not as single words. To also facilitate analysis of motifs in cWords, the most significant words are clustered into motifs. The algorithm developed for word clustering is based on the UPGMA algorithm [32]. In this implementation of UPGMA, association of two words is inferred by ungapped local alignment. An alignment of two words is scored by the number of matches minus the number of mismatches. The highest scoring ungapped alignment is found and the score is normalized dividing by the length of the shortest word to control for score biases when comparing words of different lengths. This score is used for clustering.

## Abbreviations

AGO: Argonaute protein; A549: Human lung cancer cell line; CDS: Coding sequences; DLD-1: Human colon cancer cell line; DNA: Deoxyribonucleic acid; ES index: Enrichment specificity index; FDR: False discovery rate; HCT116: Human colon cancer cell line; HEK293: Human embryonic kidney cell line; HeLa: Human cervical cancer cell line; IP: Immunoprecipitation; mRNA: Messenger RNA; miRNA: microRNA; RISC: RNA induced silencing complex; RNA: Ribonucleic acid; RNA-BP: RNA binding protein; RNAi: RNA interference; RNP: Ribonucleoprotein; siRNA: Small interfering RNA; TOV21G: Human ovary cancer cell line; UPGMA: Unweighted pair group method using arithmetic averages; UTR: Untranslated region; 3′UTR: 3′ end untranslated region

## Competing interests

The authors declare that they have no competing interests.

## Authors’ contributions

AJ originally developed the statistical framework that the current version of cWords is based supervised by AK. SHR and AK developed new methods to approximate and optimize statistics in the current version and SHR created the web server and is the maintainer of the code and web server of the current version. All authors participated in preparation of the manuscript and have approved the final draft.

## Supplementary Material

Additional file 1 Supplementary methods. Click here for file
