# Supplementary material for: cWords - systematic microRNA regulatory motif discovery from mRNA expression data
Source: Silence. 2013 May 20;4:2. doi: 10.1186/1758-907X-4-2 (PMC3682869; doi:10.1186/1758-907X-4-2)
Supplement: Additional file 1 — Supplementary methods. [file 1758-907X-4-2-S1.docx]

## Supplementary methods

### Multiple hypotheses testing and False Discovery Rate

For inference and multiple hypothesis testing we use the Benjamini-Hochberg method [1] and assume that the *p*-values (calculated in Equation 1 in the main text) assigned to each word are independent. We consider ordered *p*-values of $m$ words $p_{1},p_{2,\ldots,}p_{m}$ and for a given false discovery rate $q$ we find the largest $k$ for which

$$p_{k}\leq\frac{k}{m}q$$

In order to calculate the False Discovery Rate *FDRw* of a word *w*, we find its rank, $k$, and we calculate the smallest *FDR* for which all *p*-values at rank $k$ and higher are not rejected, we refer to this as $q(k)$*.*

$$q_{(k)}=p_{(k)}\frac{m}{k}$$

### Comparative study

miReduce and cWords were run on words of lengths 6, 7, and 8. By default Sylamer disregards 1-shift or 2-shift repeats in the sequences. In order to test the methods on fair terms, this was switched off (–r2-off). The rankings were constructed in different ways for the 3 methods. MiReduce reports a partial ranking and iteratively adds one word, and parameter, at a time; thus, gradually increasing the complexity of the model. For Sylamer to perform as well as possible we ran it with a bin size of 1 (option -grow 1), such that the background model would be as precise as possible, at the cost of longer execution time. Sylamer returns a list of *p*-values computed from the hyper-geometric distribution converted into log-scores for each word and we take the maximum across these, for positively correlated words, and the minimum for negatively correlated. Words were ranked by these scores.

We used a broad set of microarray experiments to compare the three methods; differential expression levels were measured over two conditions. The experiments were either cell lines transfected by miRNA versus mock transfection, or inhibition of miRNA vs. control. Of the 19 experiments, 18 were miRNA transfection and 1 miRNA inhibition. Two experiments where miR-1 and miR-124 were transfected in HeLa cells (Lim et al [2]). Nine experiments where HeLa cells were transfected by either miR-7, miR-9, miR-122, miR-128, miR-132, miR-133a, miR-142, miR-148b or miR-181a (Grimson et al [3]), five experiments were miR-34a transfections in different tissues (A549, DLD-1, HCT166, HeLa and TOV21G [4]), a transfection experiment of miR-146a ([5]), a transfection experiment of miR-449 ([6]) and a miR-21 knock-down experiment (Frankel et al [7]).

3’UTR sequence data was obtained from ENSEMBL, release 49; for each ENSEMBL gene ID the longest sequence was selected. miRBase version 19 annotation was used to define the seed regions.

### Data used in miR-9 and miR-128 over-expression analysis

miR-9 and miR-128 expression data was the same used in the comparative study [3]. MiRNA and mRNA sequence data: coding regions from the hg19 assembly were downloaded from University of California Santa Cruz (UCSC) table browser ([8]) group ”Genes and Gene Prediction Tracks” track ”Ensembl Genes” release GRCh37 ([9]). 3’UTRs were downloaded from Biomart (Ensembl) release 49. For both coding regions and 3’UTRs the longest corresponding sequence was chosen for each Ensembl Gene ID.

### Data used in analysis of Argonaute binding in HEK293 cells

Expression data measuring mRNA expression in immunoprecipitated (IP) Ago ribonucleoprotein particles (RNPs) relative to background mRNA expression in HEK293 cells was obtained from the supplementary material of a previous study [10]. This dataset is an average over Argonaute IP experiments done for each of the four Argonaute (AGO1-4) proteins. HEK293 miRNA expression data was obtained from a miRNA expression database for the cell line identifier ”Kidney-embryo-HEK293-exp” [11].

### Data used in analysis of siRNA transfections

Microarray datasets measuring mRNA expression changes, relative to mock transfection, in HeLa cells after transfection of unmodified and 2’-O-methyl modified siRNAs targeting *pik3ca*, *prkce* and *vhl* were obtained from the Gene Expression Omnibus (GEO): PIK3CA-2629-a [GEO:GSM134317], PIK3CA-2629-AS pos2 modified-a [GEO:GSM134318], PRKCE-1295-a [GEO:GSM134323], PRKCE-1295-AS pos2 modified-a [GEO:GSM134324], VHL-2651-a [GEO:GSM134325], VHL-2651-AS pos2 modified-a [GEO:GSM134326]. Genes not expressed, or only expressed at low levels, were excluded from the analysis. This was done by selecting genes with expression level higher than the median expression intensity, measured for each individual microarray. The siRNA sequences and reverse complementary seeds in are shown in Table S1.

| **Table S1. siRNA guide strand sequences and seed regions** | | |
| --- | --- | --- |
| **siRNAs** | **Sequence** | **Rev. compl. of seed** |
| PIK3CA-2629 | UGGCUUUGAAUCUUUGGCC | aCCGAAAC |
| PRKCE-1295 | UGAGGACGACCUAUUUGAG | aCUCCUGC |
| VHL-2651 | CAGAACCCAAAAGGGUAAG | gUCUUGGG |

Sequences of siRNAs used in the experiment and the reverse complement of the seed region. Positions in the siRNAs annotated with lowercase are independent from the actual sequence of the siRNA, analogous with position 1 in a miRNA seed region.

| **Table S2. Comparison of 7mer seed site ranks** | | | | | | |  |
| --- | --- | --- | --- | --- | --- | --- | --- |
|  | ***7mer Seed site ranking*** | | | ***Run-time (minutes:seconds)*** | | | |
|  | **cWords^†^** | **miReduce** | **Sylamer** | **cWords^†^** | **miReduce** | **Sylamer** | |
| **miR-21^*^** | 1 | 1 | 12 | 6:03 | 12:19 | 59:11 | |
| **miR-133a** | 2 | 2 | 2 | 6:12 | 9:06 | 50:42 | |
| **miR-9** | 1 | 1 | 1 | 5:14 | 15:33 | 50:45 | |
| **miR-7** | 1 | 1 | 1 | 4:50 | 13:10 | 50:54 | |
| **miR-1** | 1 | 1 | 1 | 4:00 | 9:17 | 38:14 | |
| **miR-132** | 1 | 1 | 1 | 5:09 | 14:30 | 50:18 | |
| **miR-128** | 1 | 1 | 1 | 2:42 | 14:38 | 50:16 | |
| **miR-142** | 1 | 1 | 1 | 5:45 | 11:11 | 50:06 | |
| **miR-124** | 1 | 1 | 1 | 3:53 | 8:33 | 38:05 | |
| **miR-122** | 1 | 1 | 1 | 2:32 | 17:26 | 48:22 | |
| **miR-148** | 1 | 1 | 1 | 4:54 | 11:13 | 51:25 | |
| **miR-181** | 1 | 1 | 1 | 5:29 | 13:24 | 51:05 | |
| **miR-34a (A549)** | 1 | 1 | 1 | 4:27 | 11:17 | 38:30 | |
| **miR-34a (DLD-1)** | 1 | 1 | 2 | 4:55 | 9:45 | 38:38 | |
| **miR-34a (HCT116)** | 1 | 1 | 2 | 3:47 | 9:25 | 38:10 | |
| **miR-34a (HeLa)** | 1 | 1 | 3 | 3:47 | 13:12 | 38:17 | |
| **miR-34a (TOV21G)** | 1 | 1 | 1 | 4:47 | 9:49 | 38:37 | |
| **miR-449** | 1 | 1 | 1 | 5:47 | 11:17 | 57:54 | |
| **miR-146a** | 1 | 1 | 4 | 6:08 | 12:07 | 59:05 | |
| **Summary^1^** | 1 | 1 | 1 | 4:45 | 11:57 | 47:18 | |

Ranks of 7mer seed sites from running Sylamer, miReduce and cWords on 18 miRNA transfection and 1 inhibition (marked by ^*^) experiments ([3], [7], [2], [4], [5], [6] and [12]). ^†^cWords was run on 40 cores.

^1^As summary median rank and average run-time is reported.

## References

1. Benjamini Y, Hochberg Y: **Controlling the False Discovery Rate: A Practical and Powerful Approach to Multiple Testing**. *Journal of the Royal Statistical Society. Series B (Methodological)* 1995, **57**:289–300.

2. Lim LP, Lau NC, Garrett-Engele P, Grimson A, Schelter JM, Castle J, Bartel DP, Linsley PS, Johnson JM: **Microarray analysis shows that some microRNAs downregulate large numbers of target mRNAs**. *Nature* 2005, **7027**:769–773.

3. Grimson A, Farh KK-H, Johnston WK, Garrett-Engele P, Lim LP, Bartel DP: **MicroRNA Targeting Specificity in Mammals: Determinants beyond Seed Pairing**. *Molecular Cell* 2007, **27**:91–105.

4. He L, He X, Lim LP, De Stanchina E, Xuan Z, Liang Y, Xue W, Zender L, Magnus J, Ridzon D, Jackson AL, Linsley PS, Chen C, Lowe SW, Cleary MA, Hannon GJ: **A microRNA component of the p53 tumour suppressor network**. *Nature* 2007, **447**:1130–1134.

5. Crone SG, Jacobsen A, Federspiel B, Bardram L, Krogh A, Lund AH, Friis-Hansen L: **microRNA-146a inhibits G protein-coupled receptor-mediated activation of NF-κB by targeting CARD10 and COPS8 in gastric cancer**. *Mol. Cancer* 2012, **11**:71.

6. Bou Kheir T, Futoma-Kazmierczak E, Jacobsen A, Krogh A, Bardram L, Hother C, Grønbæk K, Federspiel B, Lund AH, Friis-Hansen L: **miR-449 inhibits cell proliferation and is down-regulated in gastric cancer**. *Mol. Cancer* 2011, **10**:29.

7. Frankel LB, Christoffersen NR, Jacobsen A, Lindow M, Krogh A, Lund AH: **Programmed cell death 4 (PDCD4) is an important functional target of the microRNA miR-21 in breast cancer cells**. *J. Biol. Chem.* 2008, **283**:1026–1033.

8. Rhead B, Karolchik D, Kuhn RM, Hinrichs AS, Zweig AS, Fujita PA, Diekhans M, Smith KE, Rosenbloom KR, Raney BJ, Pohl A, Pheasant M, Meyer LR, Learned K, Hsu F, Hillman-Jackson J, Harte RA, Giardine B, Dreszer TR, Clawson H, Barber GP, Haussler D, Kent WJ: **The UCSC Genome Browser database: update 2010**. *Nucleic Acids Res.* 2010, **38**:D613–619.

9. Flicek P, Amode MR, Barrell D, Beal K, Brent S, Carvalho-Silva D, Clapham P, Coates G, Fairley S, Fitzgerald S, Gil L, Gordon L, Hendrix M, Hourlier T, Johnson N, Kähäri AK, Keefe D, Keenan S, Kinsella R, Komorowska M, Koscielny G, Kulesha E, Larsson P, Longden I, McLaren W, Muffato M, Overduin B, Pignatelli M, Pritchard B, Riat HS et al: **Ensembl 2012**. *Nucleic Acids Research* 2012, **40**:D84–D90.

10. Landthaler M, al et: **Molecular characterization of human Argonaute-containing ribonucleoprotein complexes and their bound target mRNAs.** *RNA* 2008.

11. Landgraf P, al et: **A Mammalian microRNA Expression Atlas Based on Small RNA Library Sequencing.** *Cell* 2007, **129**:1401–1414.

12. Linsley PS, Schelter J, Burchard J, Kibukawa M, al et: **Transcripts targeted by the microRNA-16 family cooperatively regulate cell cycle progression.** *Molecular Cell Biology* 2007, **27**:2240–2252.
